# Validity and reliability of Nike + Fuelband for estimating physical activity energy expenditure

**DOI:** 10.1186/s13102-015-0008-7

**Published:** 2015-06-30

**Authors:** Wesley J. Tucker, Dharini M. Bhammar, Brandon J. Sawyer, Matthew P. Buman, Glenn A. Gaesser

**Affiliations:** 1 Healthy Lifestyles Research Center, School of Nutrition and Health Promotion, Arizona State University, 550 N Third St., Phoenix, AZ 85004 USA; 2 Institute for Exercise and Environmental Medicine, Texas Health Presbyterian Hospital Dallas and UT Southwestern Medical Center Dallas, Dallas, TX USA; 3 Departments of Biology and Kinesiology, Point Loma Nazarene University, San Diego, CA USA

**Keywords:** Physical activity, Accelerometers, Measurement, Energy expenditure, SenseWear armband

## Abstract

**Background:**

The Nike + Fuelband is a commercially available, wrist-worn accelerometer used to track physical activity energy expenditure (PAEE) during exercise. However, validation studies assessing the accuracy of this device for estimating PAEE are lacking. Therefore, this study examined the validity and reliability of the Nike + Fuelband for estimating PAEE during physical activity in young adults. Secondarily, we compared PAEE estimation of the Nike + Fuelband with the previously validated SenseWear Armband (SWA).

**Methods:**

Twenty-four participants (*n* = 24) completed two, 60-min semi-structured routines consisting of sedentary/light-intensity, moderate-intensity, and vigorous-intensity physical activity. Participants wore a Nike + Fuelband and SWA, while oxygen uptake was measured continuously with an Oxycon Mobile (OM) metabolic measurement system (criterion).

**Results:**

The Nike + Fuelband (ICC = 0.77) and SWA (ICC = 0.61) both demonstrated moderate to good validity. PAEE estimates provided by the Nike + Fuelband (246 ± 67 kcal) and SWA (238 ± 57 kcal) were not statistically different than OM (243 ± 67 kcal). Both devices also displayed similar mean absolute percent errors for PAEE estimates (Nike + Fuelband = 16 ± 13 %; SWA = 18 ± 18 %). Test-retest reliability for PAEE indicated good stability for Nike + Fuelband (ICC = 0.96) and SWA (ICC = 0.90).

**Conclusion:**

The Nike + Fuelband provided valid and reliable estimates of PAEE, that are similar to the previously validated SWA, during a routine that included approximately equal amounts of sedentary/light-, moderate- and vigorous-intensity physical activity.

## Background

Self-reported physical activity from national physical activity surveillance system surveys reveal that only 45–50 % of US adults meet the recommended 150 min of physical activity per week [1]. However, objective measures for assessing physical activity levels indicate that adherence to these guidelines, including the recommendation that physical activity should be performed in episodes of at least 10 min, is less than 5 % [2]. This disparity in the estimation of physical activity and subsequent energy expenditure (EE) levels among subjective and objective measures emphasizes the need for accurate, reliable and convenient assessment methods of physical activity among free-living adults.

Portable accelerometers were developed to objectively measure physical activity and energy expenditure during free-living and structured physical activity [3–5]. The SenseWear Armband (SWA) (Bodymedia, Pittsburgh, PA) is a wireless, non-invasive physical activity monitor worn on the upper posterior of the left arm that has been previously validated for energy expenditure during free-living and structured physical activity with good levels of agreement with criterion measures [6–9]. Waist-worn accelerometers such as the Tritac 3D, CSA, Actitrac, Biotrainer and Actigraph have shown good reliability and agreement with criterion measures for estimation of EE [5, 10–12]. Wrist-worn accelerometers such as ActiWatch, GENEA, Vivago and Jawbone UP have also been shown to accurately predict EE during physical activity [13–16].

The Nike + Fuelband (Nike Inc., Beaverton, OR), released in 2012, is another device that allows users to track physical activity and estimate physical activity energy expenditure (PAEE) [17]. The Nike + Fuelband is a wireless, non-invasive, wrist-worn physical activity monitor with a triaxial accelerometer. Proprietary algorithms incorporate raw accelerometer counts with demographic characteristics such as age, height, weight and sex to estimate PAEE. We are aware of only one published report on the validity of the Nike + Fuelband for estimating EE [16]. In this study, the Nike + Fuelband produced good agreement at the group level in comparison to indirect calorimetry-measured total energy expenditure (TEE). However, the correlation between individual TEE estimates from the Nike + Fuelband and indirect calorimetry was relatively low (0.346), and the Nike + Fuelband exhibited proportional systematic bias. Furthermore, this study did not report the reliability of the Nike + Fuelband [16].

The primary purpose of this study was to determine the validity and reliability of the Nike + Fuelband for estimating PAEE in young men and women during a 60-min semi-structured physical activity routine. For comparison purposes we also had participants simultaneously wear the previously validated SenseWear Armband (SWA). We hypothesized that the Nike + Fuelband would provide valid and reliable estimates of PAEE and that these estimates would not be different from those obtained from the SWA.

## Methods

### Participants

Twenty-four, healthy, non-smoking adults (*n* = 24) were recruited via emails, listservs and flyers as part of a larger study that assessed energy expenditure of adults performing various physical activity routines (NIH R01 HL 091006). Participants were excluded if they smoked or failed to meet the Physical Activity Readiness Questionnaire (PAR-Q) requirements. Participant characteristics are listed in Table 1. This study was approved by the Arizona State University Institutional Review Board (IRB #: 0808003159) and all participants provided written informed consent prior to participation. At the initial visit, height and weight were measured. Standing height (cm) was measured to within 0.1 cm against a wall-mounted stadiometer (Seca, Germany). Body weight (kg) was measured using a calibrated electronic scale (Life Measurement Instruments, CA, USA).

**Table 1:** Participant Characteristics

*N*	24
Sex (Male, Female)	11, 13
Age (years)	28.4 ± 7.8
Height (cm)	172.7 ± 10.5
Weight (kg)	71.5 ± 16.8
Body Mass Index (kg · m^−2^)	23.8 ± 3.9
All data displayed as Mean ± SD.	

### Study protocol

On two separate occasions, participants performed a 60-min semi-structured physical activity routine in a climate-controlled laboratory (22 °C). Prior to the physical activity routine, participants rested in a seated position for 5 min and stood upright for 5 min. Thereafter participants performed, in random order, 12 activities selected from a list established using the metabolic equivalents (METs) from the Compendium of Physical Activities [18]. The randomization was designed to ensure that the routine included 4 sedentary/light-intensity activities, 4 moderate-intensity activities, and 4 vigorous-intensity activities. The list included one sedentary activity (<1.5 METs: sitting), seven light-intensity activities (1.5- < 3 METs: ironing clothes; typing; washing dishes; walking at 2.4 km∙h^−1^; arm ergometry (12.5 W); loading/unloading boxes; basketball bounce pass), ten moderate-intensity activities (3- < 6 METs: sweeping; vacuuming; walking at 4 km∙h^−1^; walking at 5.6 km∙h^−1^; resistance exercise circuit of squats, shoulder abduction, bicep curls, shoulder adduction and shoulder press with 5 kg (females) or 10 kg (males) dumbbells; cycle ergometery (~15 ml O_2_/kg/min); rowing ergometry (~15 ml O_2_/kg/min); self-paced walking on level floor that included a 1-flight stair climb approximately every min; simulated golf; basketball dribble), and six vigorous-intensity activities (≥6 METs: jogging at 8 km∙h^−1^; jogging at 9 km∙h^−1^; ascending and descending stairs; cycle ergometry (~23 ml O_2_/kg/min), rowing ergometry (~23 ml O_2_/kg/min); walking at 4.8 km∙h^−1^ with 10 % incline). Each activity lasted 4 min 50 s with a 10-s transition between activities. Although the selection and sequence of activities was randomized between participants, the two semi-structured routines performed by each participant on separate occasions were identical within participants and completed at approximately the same time of day. Participants were instructed to not consume any food for 3 h prior to each visit.

### Instruments

The Nike + Fuelband (Nike Inc., Beaverton, OR) was initialized according to manufacturer instructions with participant characteristics (i.e., age, sex, height, and weight) entered to produce predicted PAEE (kcal) estimates. Data output provided in the Nike + Software reflects total kcal for PAEE and does not include minute-by-minute data. The Nike + Fuelband was worn on the left wrist for all participants. The SenseWear armband (SWA; Model: WMS, Bodymedia, Pittsburgh, PA, USA) is a wireless, non-invasive monitor that contains a triaxial accelerometer and four heat sensors [19]. Data from each sensor are incorporated into proprietary algorithms to predict PAEE (SenseWear Professional Software 7.0). The SWA was worn on the upper posterior aspect of the left arm for all participants in accordance with manufacturer recommendations.

Each participant was also fitted with a lightweight, portable metabolic measurement system (Oxycon Mobile, Carefusion, Yorba Linda, CA, USA) (OM) that has been validated against the Douglas Bag Method [20]. Pulmonary ventilation and gas exchange were measured breath-by-breath for determination of oxygen uptake (VO_2_) and carbon dioxide production (VCO_2_), which were used to compute energy expenditure (kcal/min) [21]. Mean resting EE (REE) for each participant was estimated using the final 2 min (min 4 and 5) of the 5-min seated rest period prior to beginning the physical activity routine. To obtain PAEE for the OM, each individual’s REE was subtracted from TEE measured during the 60-min physical activity routine.

Since the version of the Nike + Fuelband we used did not have a timestamp feature, we determined PAEE by recording the kcal reading from the display on the device at the start and end of the physical activity routine. PAEE outputs that appear on the Nike + Fuelband device itself reflect 1-min epochs [22] but the absence of a software timestamp feature at the time of the study allowed us to only obtain total PAEE and not assess PAEE during individual activities. Total PAEE during the 60-min routine for the SWA was automatically calculated by the SenseWear Professional 7.0 Software. PAEE provided by the SWA was also observed in 1-min epochs and the timestamp feature was used to denote the start and finish of the physical activity routine. The exact start and stop times for each physical activity session were synchronized with the start of a new min as related to the time of day and recorded in the OM to synchronize with the SWA estimates.

### Statistical analysis

Statistical analyses were conducted using SPSS 21 (IBM Corporation, Armonk, NY, USA) with significance set at *P* < 0.05. Descriptive variables are presented as Mean ± SD. To assess validity of the Nike + Fuelband and SWA for estimation of PAEE we calculated intraclass correlation coefficients (ICC) for absolute agreement between each device and the OM from data collected during the first physical activity session. Standard conventions for interpreting good, moderate, and poor levels of agreement (validity) or reliability (test-retest) from the ICC statistic were used [23, 24]. To explore possible bias in terms of overestimation or underestimation on the Nike + Fuelband and the SWA, Bland-Altman Plots were constructed to compare against the OM, and limits of agreement were calculated as the mean difference between devices ± 2 SD [25].

Overestimation or underestimation of mean PAEE by each device was considered significant if the 95 % Confidence Intervals (95 % CIs) did not include 0. The 95 % CIs provide a stricter criterion for overestimation or underestimation than the 2 SD displayed in the Bland-Altman plots. Taken together, the ICCs for absolute agreement, the mean PAEE with 95 % CIs, and the Bland-Altman Plots provide evidence for criterion validity. As an additional measure of validity, mean absolute percent error (MAPE) for estimation of PAEE was assessed in both Nike + Fuelband and SWA:

$$ \mathrm{Mean}\;\mathrm{Absolute}\;\mathrm{Percent}\;\mathrm{Error}=\left[\mathrm{Device}\left(\mathrm{kcal}\right)\hbox{-} \mathrm{O}\mathrm{M}\left(\mathrm{kcal}\right)\right]/\mathrm{O}\mathrm{M}\left(\mathrm{kcal}\right)\ast 100 $$

To compare our results with those of Lee et al. [16] we also performed equivalence testing by comparing the 90 % CI of the mean of the PAEE estimate of each device with a 10 % equivalence zone defined as the PAEE range equal to the criterion measure (OM) mean ± 10 %, and Pearson product-moment correlations between individual PAEE estimates from the OM and each physical activity monitor. Test-retest reliability was assessed by two-way, random effects single measure intraclass correlation (ICC) between visit 1 and visit 2. The ICC is a better indicator of stability over time than the Pearson product–moment correlations as it assesses both variance explained by individuals and mean differences over time [26]. Finally, a paired T-test was conducted between visit 1 and visit 2 for each device to assess internal stability.

## Results

Participants spent 35 % of their routine in sedentary/light-intensity activity, 39 % in moderate-intensity activity and 26 % in vigorous-intensity activity using MET cutoffs based on VO_2_ measurements from the Oxycon Mobile. Gross VO_2_ during the entire physical activity routines averaged 15.7 ± 1.6 ml · kg^−1^ · min^−1^.

Both Nike + Fuelband and SWA produced estimates of PAEE that were not different from OM (Table 2). Mean absolute percent errors for PAEE estimates were similar between Nike + Fuelband (16 ± 13 %) and SWA (18 ± 18 %) compared to the criterion (Table 2). The ICCs for absolute agreement in EE estimation were moderate to good for both devices compared to OM with ICCs ranging from 0.61 to 0.77 (Table 2). The ICC results were consistent with moderate to good Pearson correlations between OM and Nike + Fuelband (*r* = 0.77) and OM and SWA (*r* = 0.61) (Table 2).

**Table 2:** Validity and reliability of Nike + Fuelband and SenseWear Armband (SWA) for estimation of physical activity energy expenditure (PAEE) in young adults

**PAEE by device (kcal ± SD)**	
Oxycon Mobile^a^	243 ± 67
Nike + Fuelband	246 ± 67
SWA	238 ± 57
**PAEE difference from criterion (kcal) (95 % CIs)**	
Nike + Fuelband – Oxycon Mobile^a^	3 (−16, 23)
SWA – Oxycon Mobile^a^	−5 (−30, 20)
**Mean absolute percent error (% ± SD) for PAEE**	
Nike + Fuelband	16 ± 13
SWA	18 ± 18
**Correlation with Oxycon Mobile for PAEE**	
Nike + Fuelband	0.77*
SWA	0.61*
**ICC Validity against Oxycon Mobile** ^**b**^	
Nike + Fuelband	0.77
SWA	0.61
**ICC Test-retest reliability** ^**b**^	
Oxycon Mobile^a^	0.94
Nike + Fuelband	0.96
SWA	0.90

*CIs* confidence intervals, *PAEE* physical activity energy expenditure, *SD* standard deviation, *SWA* sensewear armband

^a^denotes that resting energy expenditure was subtracted from this estimate to obtain PAEE

^b^denotes that test-retest reliability and absolute agreement were assessed by Two-way Random Effects ANOVA for Single Measures

*denotes correlation is significant at the *P* < 0.05 level (2-tailed)

**Fig. 1:**
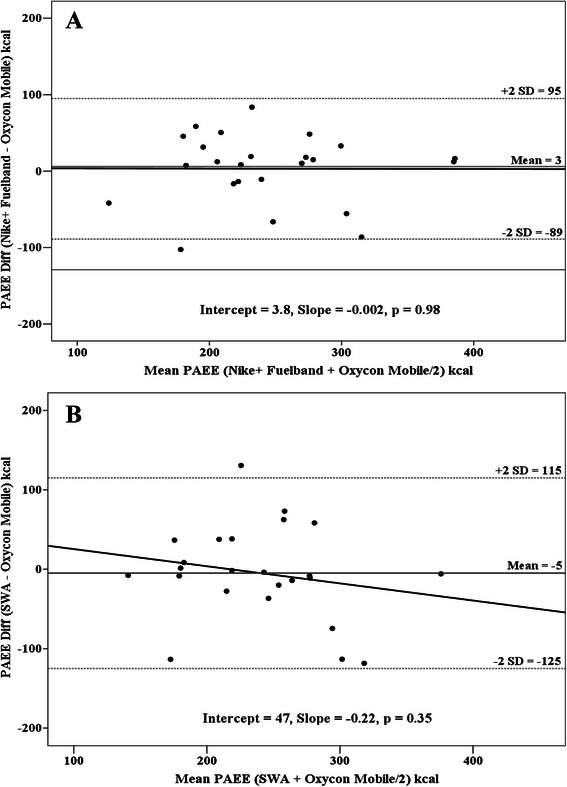
**a** Bland-Altman plot between Oxycon Mobile and Nike + Fuelband for estimates of physical activity energy expenditure (PAEE). The *solid grey lines* represent the mean difference between the methods; *dashed grey lines* represent ± 2 SD. *Solid black lines* represent regression slopes. **b** Bland-Altman plot between Oxycon Mobile and SenseWear Armband (SWA) for estimates of physical activity energy expenditure (PAEE). The *solid grey lines* represent the mean difference between the methods; *dashed grey lines* represent ± 2 SD. *Solid black lines* represent regression slopes

Bland-Altman plots (Fig. 1a and b) revealed no systematic bias in PAEE estimation for the Nike + Fuelband (slope = −0.002, *p* = 0.98) or the SWA (slope = −0.22, *p* = 0.35). Examination of Shapiro-Wilk statistics, skewness and kurtosis, and visual Q-Q plots among Nike + Fuelband and SWA differences from OM revealed homoscedasticity in the MAPE.

**Fig. 2:**
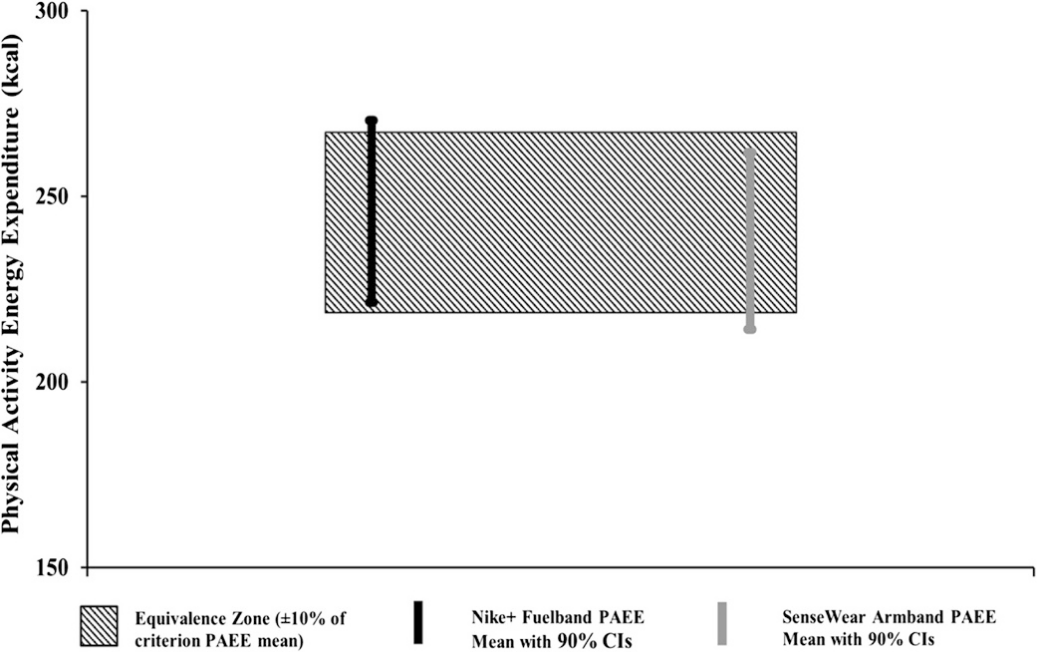
Results from 95 % equivalence testing for agreement in physical activity energy expenditure (PAEE) between Oxycon Mobile and Nike + Fuelband and SenseWear Armband (SWA) devices. *Black and white striped areas* indicate proposed equivalence zone (±10 % of the criterion value); *solid black lines* indicate 90 % CIs for estimated EE in Nike + Fuelband; *grey solid lines* indicate 90 % CIs for estimated EE in SWA

Equivalence testing showed that the Nike + Fuelband (90 % CI: 221–271 kcal) and SWA (90 % CI: 214–262 kcal) both yielded agreement that was almost within the ±10 % equivalence zone for the OM (219–267 kcal). However, neither device fit entirely within the ±10 % equivalence zone (Fig. 2).

Test-retest reliability PAEE estimates for Nike + Fuelband, SWA and OM were good in all instances (ICC range: 0.90–0.96; Table 2). A paired t-test between visit 1 and 2 for mean PAEE indicated no significant group-level differences between visits for any device (OM: *p* = 0.95, Nike + Fuelband: *p* = 0.27, SWA: *p* = 0.95).

## Discussion

Among young adults, whose 60-min routine involved spending approximately similar amounts of time in sedentary/light-, moderate- and vigorous-intensity activities, PAEE estimated by the Nike + Fuelband was not different from the criterion measure (indirect calorimetry). Furthermore, the Nike + Fuelband also demonstrated levels of validity with the OM comparable to the previously validated SWA. Our results differ from those of Lee et al. [16] who reported that for men and women similar in age to our participants, the Nike + Fuelband produced good TEE estimates but yielded a relatively low correlation with indirect calorimetry and also exhibited significant systematic bias. They used a 69-min physical activity routine similar to the one we used for our participants, and reported a MAPE of 13 % compared to our MAPE of 16 %. However, their correlation between TEE from the Nike + Fuelband and OM was only 0.35, while the correlation between PAEE from the Nike + Fuelband and OM was 0.77 for our participants. Additionally, their Bland-Altman plots revealed significant systematic bias (slope = −0.68, *p* = 0.001) whereas our Nike + Fuelband data showed no significant systematic bias (slope = −0.002, *p* = 0.98). By contrast, their equivalence testing showed that the calculated 90 % CIs for estimated TEE from the Nike + Fuelband fell well within the ±10 % equivalence zone of the OM, whereas ours for PAEE did not (although the upper 90 % CI exceeded the equivalence zone by only 4 kcal). A possible reason for the discrepancy in findings could be that we assessed PAEE, whereas Lee et al. [16] estimated TEE by adding REE from the criterion measure (OM) to the Nike + Fuelband. However, the discrepancy in findings is difficult to explain because the physical activity routines were very similar, and the OM was used as the criterion measure in both studies.

Furthermore, in addition to providing a good estimate of PAEE during the physical activity routines, the current study also demonstrated good test-retest reliability (ICC = 0.96) between visits for the Nike + Fuelband. It is important to note that our reliability estimates are not biased by using only steady-state data that would tend to enhance agreement with the OM. In contrast, the only other published report on the Nike + Fuelband did not report on the reliability of the Nike + Fuelband [16]. Our results for the Nike + Fuelband appear to be more internally consistent, as all statistical procedures used to assess validity and reliability of the Nike + Fuelband produced congruent results. In addition, the findings from this study reveal that the Nike + Fuelband provides similar criterion validity in the estimation of energy expenditure during physical activity as other previously validated wrist-worn accelerometers such as the GENEA, Vivago and Jawbone UP [14–16].

A strength of our study is the inclusion of multiple statistical procedures for assessment of validity and is consistent with the Guidelines for Reporting Reliability and Agreement Studies (GRRAS) [27]. Furthermore, it is important to note that the Nike + Fuelband achieved good validity despite performing multiple free-living activities during the 60-min physical activity routine. Accelerometers are typically more accurate for activities that include walking than free-living “lifestyle” activities [5, 10, 28]. Accelerometers such as the SWA have been reported to underestimate EE for physical activities such as cycling, incline walking and running [4, 7, 29]. In a 120-min routine that included a variety of lifestyle tasks and sporting activities, the SWA significantly overestimated EE in moderate-intensity physical activity and underestimated EE in vigorous-intensity physical activity [30]. Lastly, as was highlighted earlier in the discussion, this study is the first to report test-retest reliability for the Nike + Fuelband. This is essential as both agreement and consistency are vital in the validation of measures.

A possible limitation of our study is that we used only a 5-min seated rest period (min 4 and 5 were averaged) to establish REE. A recent study by Cunha et al. [31] revealed that a 10-min acclimation period while wearing the face mask, followed by a 30-min resting and 5-min assessment period may be required to accurately assess REE. Based on the findings of Cunha et al. [31], the difference between a 5 and 30-min assessment of REE can result in the overestimation of REE by 0.20–0.25 kcal/min which would amount to a 12–15 kcal underestimation of PAEE for the criterion measure in our study. Another limitation of our study is that we were unable to assess the accuracy of the Nike + Fuelband for specific activities. However, our study included a variety of activities spanning a wide range of intensities, and included non-steady-state periods during transitions from one activity to another. This may better reflect free-living conditions of adults, and, as suggested by Lee et al. [16], may have more ecological value with regard to the intended use of activity monitors such as the Nike + Fuelband. While it is important to understand the degree of accuracy for individual activities, previous research has shown that accelerometer-based activity monitors are able to detect TEE or PAEE fairly accurately despite large underestimation or overestimation of EE for certain activities such as cycling, incline walking, and activities of daily living [7, 19, 30], possibly due to underestimations and overestimations canceling each other out during extended periods of physical activity. Also, it should be noted that although we used free-living activities extensively in our physical activity protocol, the activities were performed in a controlled, structured laboratory environment and thus does not truly represent free-living activity. Furthermore, estimation and tracking of free-living PAEE with physical activity monitors is largely variable at both an inter- and intra-individual level due to error in the classification of physical activity type and intensity [32]. John et al. [32] attempted to overcome this limitation by using a novel, multi-sensor Integrated Physical Activity Measurement System (IMS) that receives data input from 2 accelerometers (wrist and hip-worn), 2 piezoelectric sensors on the torso and an ultraviolet radiation sensor to obtain contextual information (indoors vs outdoors). Initial results from this multi-sensor approach suggest an improvement in recognition of activity type and intensity which could potentially improve PAEE prediction and reduce variability between and within participants under laboratory and free-living settings.

Another possible limitation is that the Nike + Fuelband and SWA were worn on the left wrist and arm respectively, the non-dominant hand for ~90 % of participants. Thus PAEE associated with ironing and vacuuming, performed with the dominant hand, may not have been adequately captured. However, these activities contributed less than 12 % of the total time of the 60-min routine.

Finally, although Nike has indicated that it will no longer manufacture the Nike + Fuelband, the company has stated that it will continue to sell and provide software support for the current Nike + Fuelband [33]. In addition, Apple Inc. recently revealed that the Nike + Fuelband technology will be available in the new Apple Watch [34]. Therefore, results of the present study may be useful for users of the Nike + Fuelband technology, whether it be with the current device or with future wrist-worn physical activity monitoring devices such as the Apple Watch.

## Conclusion

The Nike + Fuelband provided valid and reliable estimates of PAEE, that are similar to the previously validated SWA, during a 60-min semi-structured routine that included approximately equal amounts of sedentary/light-, moderate- and vigorous-intensity activity. The Nike + Fuelband may allow individuals to accurately monitor their physical activity in order to achieve physical activity goals and track EE.
